# Miliary Mesothelioma

**DOI:** 10.1002/rcr2.70141

**Published:** 2025-03-10

**Authors:** Dujinthan Jayabalan, Michaela Donaghy, Chloe Charlesworth, Edward Fysh, Y. C. Gary Lee

**Affiliations:** ^1^ Department of Respiratory Medicine Sir Charles Gairdner Hospital Nedlands Western Australia Australia; ^2^ Medical School The University of Western Australia Nedlands Western Australia Australia; ^3^ Department of Respiratory Medicine St John of God Midland Public and Private Hospitals Midland Western Australia Australia; ^4^ Institute for Respiratory Health Nedlands Western Australia Australia

**Keywords:** asbestos‐related malignancy, computed tomography (CT) imaging, haematogenous metastases, miliary mesothelioma, pleural mesothelioma

## Abstract

Miliary spread of mesothelioma is a rare but important manifestation of late‐stage disease, likely due to hematogenous dissemination. This case highlights characteristic CT imaging findings, emphasizing the need for awareness among clinicians and radiologists. Recognizing this pattern can aid in diagnosis, prognostication, and appropriate referral for palliative management.

## Graphical abstract

This case highlights characteristic CT imaging findings of miliary spread of mesothelioma, emphasising the need for awareness among clinicians and radiologists.

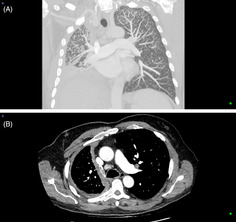

## 1 Clinical Image

A 74‐year‐old man developed right pleural mesothelioma associated with asbestos exposure from working with insulation material as an engineer. Pleural fluid analysis found epithelioid mesothelioma cells, and infection was excluded based on absent clinical signs, normal inflammatory markers, and negative cultures. He received dual immunotherapy (ipilimumab and nivolumab) and radiotherapy for tumour track metastases. Unfortunately, he developed disease progression with increased pleural tumour and lymphadenopathy on computed tomography (CT) 5 months after commencing systemic therapy. Numerous fine bilateral pulmonary deposits were noted, consistent with miliary spread of mesothelioma. He was breathless on exertion but without evidence of infection. The nodules progressed and appeared more confluent on repeat CT after 2 months. The patient was referred to palliative care for symptom‐guided supportive care.

**FIGURE 1:**
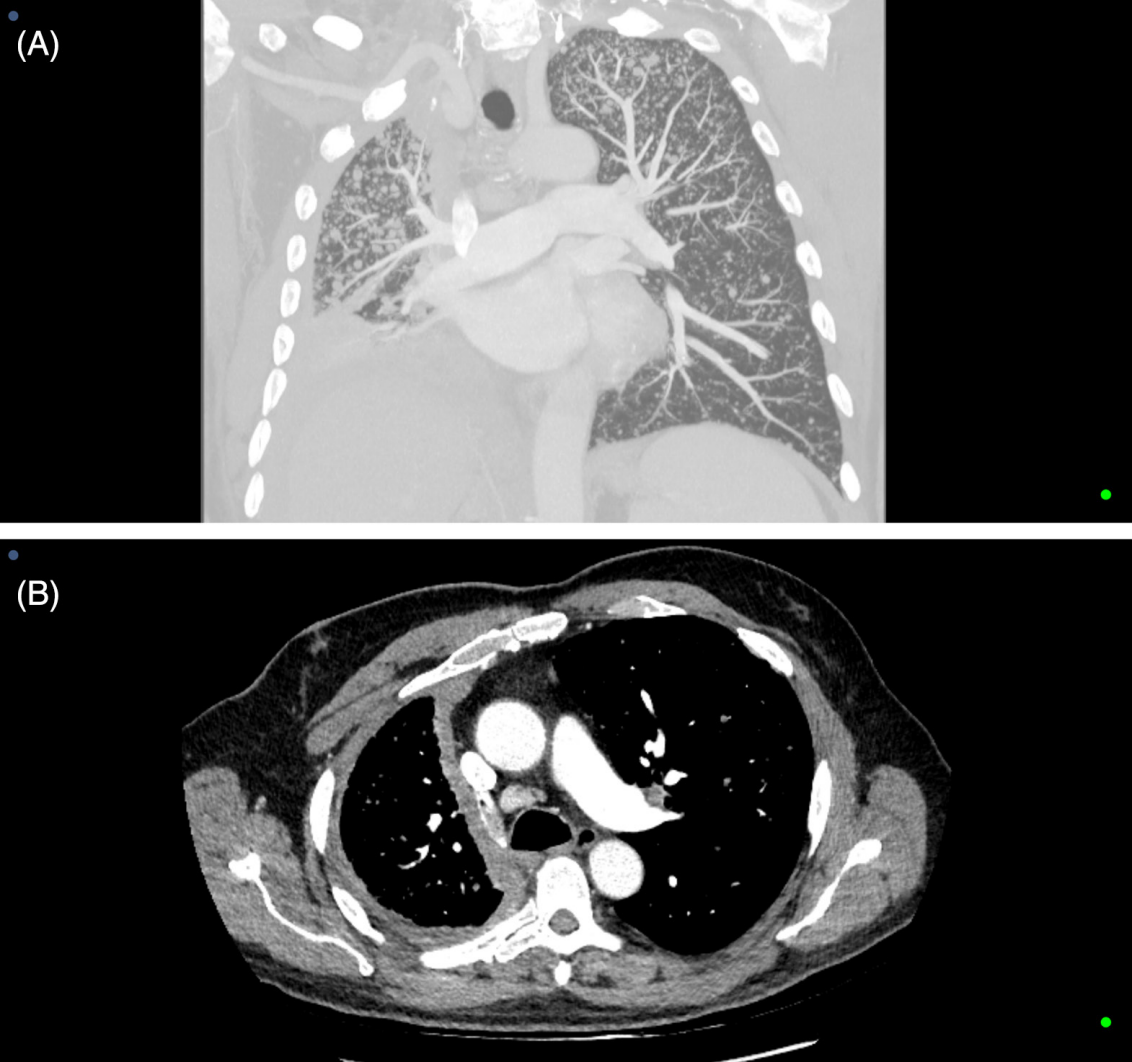
(A) Coronal computed tomography scan illustrating miliary mesothelioma. (B) Axial computed tomography scan illustrating miliary mesothelioma.

This CT image illustrates the right pleural thickening and contracted hemithorax from mesothelioma and extensive bilateral miliary lung parenchymal involvement (Figure 1A,B). Mesothelioma is generally localised to the pleural cavity [1]. Miliary lung involvement, presumed from hematogenous spread, is rare but should be considered in patients with late‐stage disease, especially with characteristic imaging [2].

## Author Contributions

The authors confirm their contribution to the paper as follows: Study conception and design: D.J. and Y.C.G.L. Patient data/information collection: D.J., M.D., C.C., and E.F. Manuscript preparation: D.J. and Y.C.G.L. Study supervision: Y.C.G.L. and E.F. All authors reviewed the results and approved the final version of the manuscript, including the authorship list.

## Consent

The authors declare that written informed consent was obtained for the publication of this manuscript and accompanying images and attest that the form used to obtain consent from the patient complies with the Journal requirements.

## Conflicts of Interest

E.F. and Y.C.G.L. are Editorial Board members of Respirology Case Reports and a co‐authors of this article. They were excluded from all editorial decision‐making related to the acceptance of this article for publication. The other authors declare no conflicts of interest.

## Data Availability

Data sharing is not applicable to this article as no new data were created or analyzed in this study.
